# Oxylipins in moss development and defense

**DOI:** 10.3389/fpls.2015.00483

**Published:** 2015-07-03

**Authors:** Inés Ponce de León, Mats Hamberg, Carmen Castresana

**Affiliations:** ^1^Departamento de Biología Molecular, Instituto de Investigaciones Biológicas Clemente Estable, Montevideo, Uruguay; ^2^Division of Physiological Chemistry II, Department of Medical Biochemistry and Biophysics, Karolinska Institutet, Stockholm, Sweden; ^3^Departamento de Genética Molecular de Plantas, Centro Nacional de Biotecnología, Consejo Superior de Investigaciones Científicas, Madrid, Spain

**Keywords:** lipoxygenases, alpha-dioxygenases, polyunsaturated fatty acids, oxylipins, moss, defense, development

## Abstract

Oxylipins are oxygenated fatty acids that participate in plant development and defense against pathogen infection, insects, and wounding. Initial oxygenation of substrate fatty acids is mainly catalyzed by lipoxygenases (LOXs) and α-dioxygenases but can also take place non-enzymatically by autoxidation or singlet oxygen-dependent reactions. The resulting hydroperoxides are further metabolized by secondary enzymes to produce a large variety of compounds, including the hormone jasmonic acid (JA) and short-chain green leaf volatiles. In flowering plants, which lack arachidonic acid, oxylipins are produced mainly from oxidation of polyunsaturated C18 fatty acids, notably linolenic and linoleic acids. Algae and mosses in addition possess polyunsaturated C20 fatty acids including arachidonic and eicosapentaenoic acids, which can also be oxidized by LOXs and transformed into bioactive compounds. Mosses are phylogenetically placed between unicellular green algae and flowering plants, allowing evolutionary studies of the different oxylipin pathways. During the last years the moss *Physcomitrella patens* has become an attractive model plant for understanding oxylipin biosynthesis and diversity. In addition to the advantageous evolutionary position, functional studies of the different oxylipin-forming enzymes can be performed in this moss by targeted gene disruption or single point mutations by means of homologous recombination. Biochemical characterization of several oxylipin-producing enzymes and oxylipin profiling in *P. patens* reveal the presence of a wider range of oxylipins compared to flowering plants, including C18 as well as C20-derived oxylipins. Surprisingly, one of the most active oxylipins in plants, JA, is not synthesized in this moss. In this review, we present an overview of oxylipins produced in mosses and discuss the current knowledge related to the involvement of oxylipin-producing enzymes and their products in moss development and defense.

## Introduction

Oxylipins are a group of structurally diverse oxygenated fatty acids present in most living organisms. In mammals, eicosanoids, notably prostaglandins, leukotrienes, and thromboxanes, are generated mainly by oxygenation of arachidonic acid (20:4) by lipoxygenases (LOXs) or cyclooxygenases (COXs), and their roles in many physiological processes including the immune response, inflammation, and hemostasis have been extensively studied (Smith et al., 2000). In flowering plants, which lack 20:4, oxylipins are produced mainly from the polyunsaturated C18 fatty acids linolenic (18:3) and linoleic (18:2) acids by oxygenations catalyzed by LOXs or α-dioxygenases (α-DOXs; Hamberg et al., 2002; Andreou et al., 2009; Mosblech et al., 2009). Plant α-DOXs and mammalian COXs have common structural and catalytic features and are functionally related (Sanz et al., 1998; Goulah et al., 2013). Non-enzymatic reactions in the presence of singlet oxygen or by free radical-mediated oxygenation can also produce oxylipins from polyunsaturated fatty acids (Mueller and Berger, 2009). In flowering plants, LOXs insert molecular oxygen primarily at either the carbon-9 (9-LOX) or carbon-13 (13-LOX) positions of 18:2 and 18:3 (Feussner and Wasternack, 2002; Mosblech et al., 2009). The resulting 9- and 13-hydroperoxides are further metabolized by secondary enzymes, notably cytochromes P-450 belonging to the CYP-74 subfamily (Lee et al., 2008). Thus, conversions catalyzed by allene oxide synthase (AOS), divinyl ether synthase (DES), or hydroperoxide lyase (HPL), lead to the formation of allene oxides, divinyl ethers, or short-chain aldehydes, respectively (Blée, 2002; Howe and Schilmiller, 2002; Andreou et al., 2009; Mosblech et al., 2009). Products formed from 9-hydroperoxy fatty acids include ketols and cyclopentenones (AOS; Hamberg, 2000; Stumpe et al., 2006a), colneleic and colnelenic acids (DES; Grechkin, 2002; Fammartino et al., 2007), and C_9_ aldehydes and oxoacids (HPL; Mita et al., 2007). Additionally, isomeric epoxy alcohols are generated in the presence of epoxy alcohol synthase (Hamberg, 1999; Lee et al., 2008). 13-Hydroperoxy acids are converted by HPL to C_6_ aldehydes and oxoacids (Matsui, 2006), by DES to etheroleic and etherolenic acids (Hamberg, 1998; Grechkin, 2002; Stumpe et al., 2008), and by AOS to unstable allene oxides (Tijet and Brash, 2002). These latter compounds undergo non-enzymatic hydrolysis to form ketols, or can be transformed into the cyclopentenone (+)-*cis*-12-oxophytodienoic acid (OPDA) in the presence of allene oxide cyclase (Wasternack, 2007). OPDA is further transformed by reduction of the ring double bond and three rounds of β-oxidation to produce the hormone jasmonic acid (JA; Wasternack, 2007). LOX-derived oxylipins are involved in several physiological processes, including fertilization, seed and root development, germination, fruit development, and senescence, and in defense responses against microbial pathogens, insects, and wounding (Howe and Schilmiller, 2002; Browse, 2005; Vellosillo et al., 2007; Kachroo and Kachroo, 2009; López et al., 2011; Wasternack and Hause, 2013).

α-DOXs catalyze the incorporation of molecular oxygen at the α-methylene carbon atom of fatty acids. Two main groups of α-DOXs have been described in flowering plants. While α-DOX1 has a substrate preference for polyunsaturated C18 fatty acids such as oleic acid, 18:2 and 18:3, the α-DOX2 isoforms can oxygenate a wider range of fatty acids including long chain (C14–22) and very-long-chain (C24–30) fatty acids (Hamberg et al., 2002, 2005; Bannenberg et al., 2009). The products of α-DOXs are chemically unstable 2(*R*)-hydroperoxy fatty acids, which either suffer spontaneous decarboxylation into the chain-shortened fatty aldehyde or are converted by reduction into the corresponding 2(*R*)-hydroxy fatty acid (Sanz et al., 1998; Hamberg et al., 1999, 2002). The α-DOX1 type of enzymes participates mainly in defense against microbial pathogens and herbivores, and senescence (Sanz et al., 1998; Obregón et al., 2001; Ponce de León et al., 2002; Hamberg et al., 2003; Vicente et al., 2012; Avila et al., 2013), while the α-DOX2 isoforms are more involved in development processes (van der Biezen et al., 1996; Bannenberg et al., 2009; Steppuhn et al., 2010; Gaquerel et al., 2012).

Bryophytes, including mosses, liverworts, and hornworts, are early divergent land plants that are phylogenetically placed between unicellular green algae and flowering plants. Mosses were the first plants to conquest land and have evolved adaptation mechanisms to tolerate extreme conditions such as desiccation and exposure to damaging UV-B radiation and to resist co-evolving pathogens and herbivores (Rensing et al., 2008). Mosses use many alternative metabolic pathways, some of which are not present in flowering plants, and probably this has allowed mosses to occupy and function in very different habitats (Rensing et al., 2007). In addition to polyunsaturated C18 fatty acids, mosses have also large amounts of polyunsaturated C20 fatty acids which are rarely present in flowering plants due to the lack of the corresponding biosynthetic enzymes (Gill and Valivety, 1997). In mosses like *Physcomitrella patens* (*P. patens*) and *Mnium cuspidotum*, 20:4 reaches up to 30% of total fatty acids (Anderson et al., 1972; Grimsley et al., 1981; Beike et al., 2014). The enzymes involved in the biosynthesis of 20:4 and eicosapentaenoic acid (20:5) have been identified and characterized in *P. patens* (Girke et al., 1998; Zank et al., 2002; Kaewsuwan et al., 2006). 20:4 and 20:5 are produced from 18:2 and 18:3, respectively, by a reaction series involving the activities of a Δ6-desaturase, a Δ6-elongase, and a Δ5-desaturase (Kaewsuwan et al., 2006). The high abundance of long and very long chain fatty acids together with the presence of oxylipins derived from 20:4 and 20:5 represent a metabolic difference between mosses and flowering plants that may provide a metabolic advantage to the adaptation capacity of mosses to severe environmental conditions (Mikami and Hartmann, 2004). LOX-derived oxylipins produced from C20 and C18 polyunsaturated fatty acids are also found in multicellular algae, where they play a role in defense responses against an algal pathogen (Bouarab et al., 2004). In unicellular algae, aldehydes derived from C20 fatty acids accumulate after wounding where they may play defensive roles (Pohnert, 2000; Pohnert and Boland, 2002). Thus, like algae, mosses have both octadecanoid and eicosanoid pathways. This review is focused on current knowledge related to oxylipins produced in mosses with a special emphasis on the role played by oxylipin-producing enzymes and their products in moss development and defense.

## Oxylipin-Producing Enzymes in *Physcomitrella patens*

**FIGURE 1:**
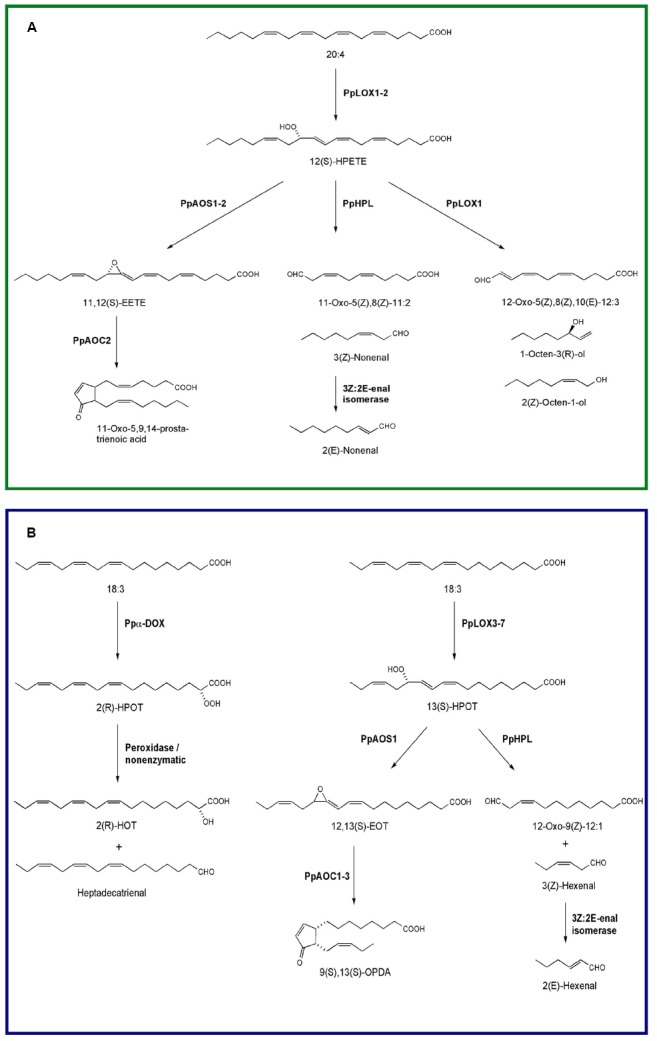
**Oxylipin biosynthesis pathways in the moss *P. patens*. (A)** Lipoxygenase-catalyzed oxygenation of arachidonic acid into 12(*S*)-HPETE and further conversions of this hydroperoxide by allene oxide synthase (PpAOS1-2), allene oxide cyclase (PpAOC2), hydroperoxide lyase (PpHPL), and 12-lipoxygenase (PpLOX1). **(B)** Oxygenation of linolenic acid into 2(*R*)-HPOT by Ppα-DOX and by lipoxygenase into 13(*S*)-HPOT. Breakdown of 2(*R*)-HPOT into 2(*R*)-HOT and heptadecatrienal and the further conversions of 13(*S*)-HPOT by allene oxide synthase (PpAOS1), allene oxide cyclase (PpAOC1-3), and hydroperoxide lyase (PpHPL) are also illustrated.

*Physcomitrella patens* is the first moss species with an available sequenced genome (Rensing et al., 2008), and several of the oxylipin-forming enzymes have been identified and biochemically characterized (Figure 1). *P. patens* has eight genes encoding lipoxygenase of which seven are functionally active *in vitro* (Anterola et al., 2009). Five are 13-LOXs (PpLOX3–PpLOX7) which use 18:3 as a substrate, while the other two are 12-LOXs (PpLOX1 and PpLOX2) and prefer 20:4 and 20:5 (Anterola et al., 2009). PpLOX3, 4, 6, and 7 can also use 18:2 as a substrate, although the activity is much higher against 18:3 (Anterola et al., 2009). PpLOX1 is an unusual bifunctional LOX that exhibit hydroperoxidase and a fatty acid chain-cleaving lyase activity (Senger et al., 2005; Anterola et al., 2009). In addition, both 12-LOXs accept C18-fatty acids as substrates yielding a broader range of oxylipins (Senger et al., 2005; Wichard et al., 2005; Anterola et al., 2009). LOX-derived 12-hydroperoxy eicosatetraenoic acid (12-HPETE) is further metabolized by the bifunctional LOX, by at least one classical hydroperoxide lyase (PpHPL; Stumpe et al., 2006b), and by two allene oxide synthases (PpAOS; Bandara et al., 2009; Scholz et al., 2012; Figure 1A). From 12-HPETE the unusual PpLOX1 produces C_8_ volatiles including (2*Z*)-octen-1-ol and 1-octen-3-ol, and 12-oxo-dodecatrienoic acid (12-ODTE; Senger et al., 2005). PpHPL transforms 12-HPETE into (3*Z*)-nonenal, which is rapidly isomerized to (2*E*)-nonenal (Stumpe et al., 2006b; Figure 1A). Experiments with labeled fatty acids in other moss species demonstrated that C_8_ volatiles are exclusively derived from C20 fatty acids, suggesting a common 12-LOX mediated biosynthetic pathway in mosses (Croisier et al., 2010). These authors have also shown that the biosynthesis of C_6_ volatiles, i.e., (3*Z*)-hexenal and hexanal, in different moss species depended on both, C18 and C20 fatty acids (Croisier et al., 2010). PpHPL is an unspecific HPL that metabolizes hydroperoxides derived from both 18:2 and 18:3, but it preferentially uses 9-hydroperoxyoctadecadienoic acid (9-HPOD) as its substrate forming (3*Z*)-nonenal and 9-oxononanoic acid *in vitro* (Stumpe et al., 2006b). Interestingly, while in the PpHPL mutant no (3*Z*)-nonenal is formed, hexanal is still produced, indicating the presence of a hexanal forming enzyme that has not been identified yet (Stumpe et al., 2006b; Scholz et al., 2012). PpAOS1 uses C18-hydroperoxides (Figure 1B) and C20-hydroperoxides (Figure 1A) as substrates yielding 12,13-epoxy-octadecatrienoic acid (12,13-EOT) or 11,12-epoxy-eicosatetraenoic acid (11,12-EETE) when 13-HPOT or 12-HPETE are metabolized, respectively, while PpAOS2 prefers C20-hydroperoxides (Bandara et al., 2009; Scholz et al., 2012). PpAOS1 can also use 9- and 13-LOX-derived hydroperoxides from 18:2 (Scholz et al., 2012). In the absence of allene oxide cyclase activity, the unstable allene oxides are further converted to ketols and racemic cyclopentenones (Stumpe et al., 2010; Hashimoto et al., 2011; Neumann et al., 2012). In the presence of PpAOC2, 11,12-EETE formed the cyclopentenone 11-oxo-5,9,14-prostatrienoic acid (11-OPTA), whereas PpAOC1, PpAOC2, and PpAOC3 metabolized 12,13-EOT into OPDA, the precursor of JA (Stumpe et al., 2010; Hashimoto et al., 2011; Scholz et al., 2012; Figure 1). Individual *P. patens* mutants lacking either PpAOC1 or PpAOC2 have similar OPDA contents compared to wild-type plants (Stumpe et al., 2010), while in the PpAOS1 mutant the synthesis of OPDA is highly impaired, indicating that PpAOS1 plays a major role in OPDA formation (Scholz et al., 2012). *P. patens* contains several putative 12-oxophytodienoic acid reductases (OPR; Breithaupt et al., 2009; Li et al., 2009), however, JA is not synthesized in this moss. It seems likely that the enzyme OPR3 responsible for JA biosynthesis is missing; indicating that only the plastidic part of the LOX pathway is present in this moss (Stumpe et al., 2010; Ponce de León et al., 2012). This is further supported by the plastidic localization of all PpLOXs, PpAOCs, and PpAOS2 (Stumpe et al., 2010; Hashimoto et al., 2011; Scholz et al., 2012). The lack of JA is not limited to *P. patens* since the liverwort *Marchantia polymorpha* (*M. polymorpha*) does not synthesize JA (Yamamoto et al., 2015), suggesting that this hormone appeared later in plant evolution. Interestingly, *P. patens* respond to JA by altering moss development (Ponce de León et al., 2012), suggesting that the downstream components are already present in basal land plants like mosses. *P. patens* contains six putative genes encoding the JA-isoleucine receptor coronatine insensitive (COI; Chico et al., 2008). It is tempting to speculate that the different *P. patens* COI-like proteins may recognize, in addition to JA, other oxylipins allowing the binding of a broader range of ligands. However, further studies are needed to understand how JA is perceived in this moss.

*Physcomitrella patens* has only a single copy of Ppα-DOX. Like α-DOX1 of *Arabidopsis thaliana* (*A. thaliana*, Hamberg et al., 2005, 2006; Machado et al., 2015), this enzyme converts fatty acids to chemically unstable 2-hydroperoxy derivatives with a substrate preference for palmitic acid (16:0), 18:3, and 18:2. 2(*R*)-hydroperoxylinolenic acid (2-HPOT) formed from 18:3 is transformed to 8,11,14-heptadecatrienal and 2-hydroxylinolenic acid (2-HOT; Figure 1B, Machado et al., 2015). In contrast to the two Pp12-LOXs, Ppα-DOX is not capable of using 20:4 as a substrate (Machado et al., 2015).

## Oxylipins in Moss Development

**FIGURE 2:**
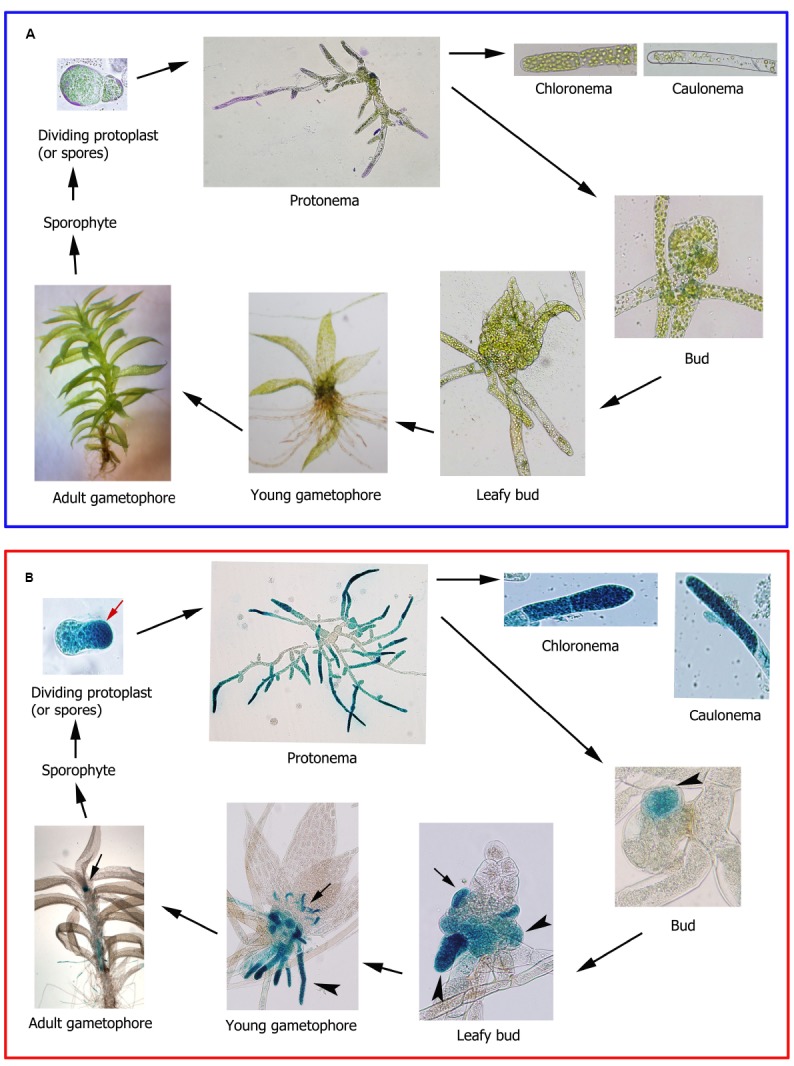
***Physcomitrella patens* life cycle. (A)** Different stages of *P. patens* life cycle. Protoplast and protonema were stained with toluidine blue for proper visualization. **(B)** Ppα-DOX-GUS expression during *P. patens* life cycle. Apical cell of a dividing protoplast is indicated with a red arrow. GUS-stained cells leading to rhizoids, rhizoid primordial and rhizoids are indicated with an arrowhead. GUS-stained axillary hairs primordial and axillary hairs are indicated with an arrow.

Mosses are land plants with a relatively simple developmental pattern with alternating haploid gametophyte and diploid sporophyte generations. The gametophyte consists of two distinct developmental stages; the juvenile filamentous protonema with chloronema and caulonema types of cells, and the adult gametophores which are leafy shoots composed of a non-vascular stem with leaves, the reproductive organs, and filamentous rhizoids (Reski, 1998; Figure 2A). The germination of a haploid spore or the division of a protoplast lead to the formation of chloronema cells with characteristic perpendicular cross walls and a high density of chloroplasts. From chloronemal filaments caulonemal cells arise subsequently with oblique cross walls and low density of chloroplasts. Branching of caulonemal cells result in new chloronemal or caulonemal cells leading to the formation of secondary chloronemal or caulonemal filaments and buds (Cove and Knight, 1993; Cove et al., 2006). Buds develop into leafy gametophores upon which the diploid sporophyte generation is formed leading to new haploid spores (Cove et al., 2006; Figure 2A). In mosses fatty acid compositions vary depending on the type of tissue. While 16:0 and 20:4 content are similar in protonemal filaments and leafy gametophores of different moss species, 18:2 and 18:3 are more abundant in gametophores and protonemal tissues, respectively (Beike et al., 2014). These metabolic differences correlate with differences in expression levels of fatty acid desaturases encoding genes (Beike et al., 2014). In maturing sporophytes of the moss *Mnium cuspidotum*, 16:0 and 20:4 are the most abundant fatty acids, while 18:2 increases when spores have matured and are ready for dispersal, reaching similar levels as 16:0 (Anderson et al., 1972). The most abundant free hydro(per)oxy fatty acids present in protonemal tissues of *P. patens* are 13-hydroperoxy linoleic acid and 12-HPETE (Stumpe et al., 2010), which can be further metabolized by the corresponding enzymes producing oxylipins with possible roles in moss development. However, the specific functions of the C18:2, C18.3, and C20:4 pathways in different tissues and during moss development are at present unknown. In *P. patens*, functional analysis of genes encoding enzymes involved in fatty acid and oxylipin biosynthesis can be performed by targeted gene disruption or single point mutation, due to its high rate of homologous recombination (Schaefer, 2002). The *P. patens* genes encoding Δ6-desaturase, Δ6-elongase, and Δ5-desaturase have been functionally characterized by targeted gene disruption, confirming their involvement in 20:4 and 20:5 production (Girke et al., 1998; Zank et al., 2002; Kaewsuwan et al., 2006). Although 20:4 and 20:5 decrease drastically in these mutants, protonema and gametophores of the knockout plants do not have a visible altered phenotype, suggesting that 20:4 and 20:5 are not necessary, or that residual amounts of these fatty acids are sufficient for normal development (Girke et al., 1998; Zank et al., 2002; Kaewsuwan et al., 2006). Some *P. patens* knockout mutants in genes encoding oxylipin-producing enzymes have also been generated. PpHPL, PpAOS1, and PpAOS2 knockout mutant have no developmental alterations (Stumpe et al., 2006b; Scholz et al., 2012), while individual PpAOC1 and PpAOC2 knockout mutants have reduced fertility, aberrant sporophyte morphology, and interrupted sporogenesis (Stumpe et al., 2010). Double PpAOC1 and PpAOC2 knockout mutants were unable to obtain suggesting that depletion of both enzymes is lethal (Stumpe et al., 2010). As mentioned previously, only the PpAOS1 mutant is highly impaired in OPDA synthesis, although the amount produced is sufficient for normal development (Scholz et al., 2012). *A. thaliana* mutants defective in oxylipin production, including OPDA- and JA-deficient mutants, and mutants in the COI-1 gene are male sterile (Browse, 2009), while a mutation in the tomato COI-1 gene renders female sterile plants (Li et al., 2004). Thus, different oxylipins participate in the development of reproductive structures, both in mosses and flowering plants. Further analysis of the different genes encoding enzymes involved in the LOX pathways are needed to understand their participation in different moss developmental processes. For example, while PpAOC1 and PpAOC2 have similar expression patterns in protonemal and gametophores tissues, PpAOC3 is preferentially expressed in protonemata tissues where it could play a more specific role (Hashimoto et al., 2011).

**FIGURE 3:**
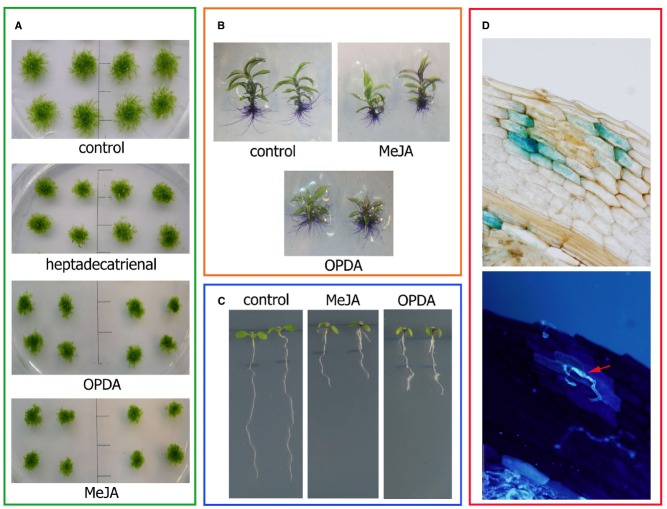
**Plant oxylipins participate in development and defense. (A)** Moss colonies showing inhibited growth compared to control plants when tissues are incubated with 50 μM Ppα-DOX-derived heptadecatrienal, 50 μM PpLOX-derived OPDA or 50 μM methyl jasmonate (MeJA). The scale bars represent 1 cm. **(B)** Moss gametophores showing reduced growth of rhizoids compared to control gametophores when tissues are incubated with 50 μM OPDA or 50 μM methyl jasmonate (MeJA). Rhizoids were stained with toluidine blue for proper visualization. **(C)** *A. thaliana* seedlings showing growth arrest of roots compared to control plants when they are incubated with 20 μM OPDA or 50 μM methyl jasmonate (MeJA). **(D)** Ppα-DOX expression in a *B. cinerea*-infected leaf showing GUS accumulation in cells surrounding infected cells (upper panel). The same leaf showing *B. cinerea* hyphae (red arrow) stained with the fluorescent dye solophenyl flavine 7GFE 500 (lower panel).

**FIGURE 4:**
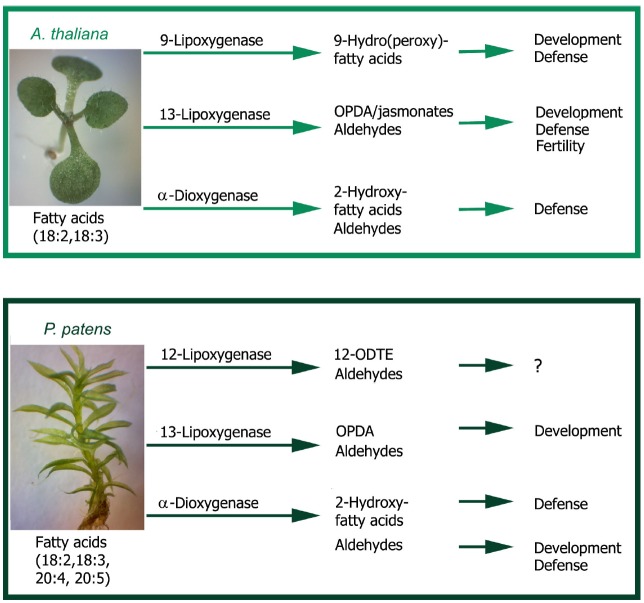
**Regulation of development and defense by plant oxylipins.** Flowering plants like *A. thaliana* have 9-lipoxygenases and 13-ipoxygenases that catalyze the oxygenation of polyunsaturated fatty acids, mainly linoleic acid (18:2) and linolenic acid (18:3), to form fatty acid hydroperoxides. The resulting hydroperoxides are further metabolized to produce a variety of oxylipins, including hydroxy fatty acids and aldehydes, which play different roles in development and defense. α-Dioxygenase utilizes 18:2 and 18:3 to form 2-hydroperoxy fatty acids that are converted to 2-hydroxy fatty acids and aldehydes, with regulatory functions in plant defense against microbial pathogens and insects. The moss *P. patens* has besides polyunsaturated C18 fatty acids, polyunsaturated C20 fatty acids and can produce a broad range of oxylipins derived from them. *P. patens* 13-lipoxygenases and α-dioxygenase utilize mainly 18:2 and 18:3 as substrates producing similar oxylipins as in flowering plants, including (+)-*cis*-12-oxophytodienoic acid (OPDA), 2-hydroxy fatty acids and aldehydes with functions in development and defense against microbial pathogens. However, jasmonates are not synthesized in *P. patens* and the 9-lipoxygenase pathway seems not to be present. In addition, 12-lipoxygenases produce oxylipins from arachidonic (20:4) and eicosapentaenoic acids (20:5), including 12-oxo-dodecatrienoic acid (12-ODTE) and aldehydes whose functions are at present unknown.

Recent studies performed with *P. patens* Ppα-DOX-GUS reporter lines have shown that Ppα-DOX is expressed during development in tips of protonemal filaments with maximum expression levels in mitotically active undifferentiated apical chloronemal and caulonemal cells (Machado et al., 2015; Figure 2B). Ppα-DOX-GUS is also highly expressed in other type of mitotically active cells, including apical cells of regenerating protoplasts (Figure 2B). The role played by Ppα-DOX-derived oxylipins in undifferentiated apical cells, which are self-renewing stem cells, needs further investigation. Interestingly, the mammalian COX-derived oxylipin PGE_2_ has sustaining effects on undifferentiation and stimulate self-renewal and proliferation (Goessling et al., 2009; Hoggatt et al., 2009), and Ppα-DOX-derived oxylipins could play similar functions. In young buds Ppα-DOX transcripts accumulate in cells leading to rhizoids and axillary hair formation suggesting that local cues present in these types of cells contribute to Ppα-DOX expression. Auxin is a good candidate since in young and adult gametophores, Ppα-DOX is expressed in auxin producing tissues, including rhizoids and axillary hairs primordial, and axillary hairs and rhizoid (Machado et al., 2015; Figure 2B). Gametophytes and sporophytes of Ppα-DOX mutant are similar to wild-type plants indicating that this enzyme is not essential for proper moss development (Machado et al., 2015). However, incubating wild-type tissues with Ppα-DOX-derived oxylipins, or overexpressing Ppα-DOX, alter *P. patens* development leading to smaller moss colonies with less protonemal tissues (Machado et al., 2015). The Ppα-DOX-derived aldehyde, heptadecatrienal, is responsible for the reduced protonemal filament growth (Figure 3A). Moss colonies are also smaller and have less protonemal tissues when moss tissues are grown in the presence of 13-LOX-derived oxylipins, including OPDA and methyl jasmonate (Ponce de León et al., 2012; Figure 3A). OPDA and jasmonate also reduce rhizoid length (Ponce de León et al., 2012), consistently with the growth arrest of *A. thaliana* seedlings and roots incubated with these oxylipins (Vellosillo et al., 2007; Mueller et al., 2008; Figures 3B,C). Interestingly, the inhibitory effect of OPDA on growth, either by OPDA application or by the generation of overexpressing MpAOC plants which produces high levels of OPDA, was also observed in the liverwort *M. polymorpha* (Yamamoto et al., 2015), suggesting a conserved response to this oxylipin among bryophytes. In contrast, JA did not affect *M. polymorpha* growth (Yamamoto et al., 2015), indicating that the growth inhibitory activity of jasmonate is not conserved among mosses and liverworts. One possible explanation is that *M. polymorpha* does not have the downstream components necessary for sensing the presence of JA. However, putative orthologs of the jasmonate ZIM-domain (JAZ) repressor and the receptor COI have been identified in *M. polymorpha* (Katsir et al., 2008; Wang et al., 2015). In addition, sequence alignment demonstrates that the binding sites are well conserved between COI orthologs of *P. patens* and *M. polymorpha* and *A. thaliana* COI (Wang et al., 2015). Further studies are needed to understand the differential response to jasmonates between different bryophytes. Mueller et al. (2008) have suggested that in flowering plants the inhibition of growth observed with OPDA is related to the inhibition of cell cycle progression. Studies in *A. thaliana* have shown that jasmonate reduces both cell number and cell size in roots and leaves (Chen et al., 2011; Noir et al., 2013). Consistently, protonemal tissues grown in the presence of heptadecatrienal have filaments with smaller caulonemal cells and abnormal cell divisions (Machado et al., 2015). Taking together, all these observations indicate that like more complex plants where oxylipins synthesized from different biochemical pathways act as regulators of development, a fine-tuning mechanism operates in order to regulate oxylipins concentrations in moss tissues for proper development. *P. patens*- and *A. thaliana*-derived oxylipins involved in plant development are schematized in Figure 4.

## Oxylipins in Moss Defense Responses to Wounding and Pathogens

In flowering plants a diversity of oxylipins are produced after wounding or pathogen attack. Different roles have been assigned to these oxylipins, including antimicrobial activities (Prost et al., 2005; Kishimoto et al., 2008), regulation of cell death (Vollenweider et al., 2000; Ponce de León et al., 2002; Hamberg et al., 2003; Montillet et al., 2005), callose deposition (Vellosillo et al., 2007; López et al., 2011), and induction of genes involved in defense (Stintzi et al., 2001; Mueller et al., 2008; Browse, 2009). In response to wounding *P. patens* produces a variety of oxylipins, including volatiles derived from 12-HPETE such as octenols (C_8_) and (3Z)-nonenal (C_9_; Senger et al., 2005; Stumpe et al., 2006b). These volatiles were also found in an oxylipin survey of 23 mosses, where the C_8_ oxylipins were the dominant volatiles in wounded mosses (Croisier et al., 2010). Interestingly, C_8_ volatiles can induce the expression of defense genes in flowering plants and increase resistance against pathogens (Kishimoto et al., 2007). Moreover, C_8_ volatiles are bioactive metabolites against arthropods (Combet et al., 2006) and C_9_ volatiles have antimicrobial activities against the fungal pathogens *Botrytis cinerea (B. cinerea)* and *Fusarium oxysporum* (Matsui et al., 2006). The moss *Dicranum scoparium (D. scoparium)* produces after wounding a diverse group of C_5_, C_6_, C_8_, and C_9_ metabolites, derived from C18 or C20 fatty acids, suggesting the presence of different LOXs and additional HPL(s), or unspecific LOXs which produce a high diversity of oxylipins (Senger et al., 2005; Croisier et al., 2010). More than 50% of 18:2, 18:3, and 20:4 available for oxylipin biosynthesis are consumed in *D. scoparium* during wounding indicating a highly efficient transformation of precursor fatty acids (Croisier et al., 2010). The dominant acetylenic fatty acid of *D. scoparium*, dicranin (9,12,15-octadecatrien-6-ynoic acid), produces after wounding the cyclopentenone dicranenone A (6,6,7,7-tetradehydro-OPDA), which has anti-feeding activity against slugs (Rempt and Pohnert, 2010). OPDA is also produced after wounding in *D. scoparium* (Rempt and Pohnert, 2010) and in *P. patens* (Scholz et al., 2012). Some of the C18:2- and C18:3-derived oxylipins produced in wounded mosses also accumulate in flowering plants after pathogen assault; this includes C_6_ volatiles which induce defense related genes, exert direct antimicrobial activity, and stimulate the accumulation of phytoalexin (Zeringue, 1992; Croft et al., 1993; Bate and Rothstein, 1998; Kishimoto et al., 2008). Thus, many of the oxylipins that accumulate in wounded moss tissues probably play a protective role against pathogens. However, only few studies related to oxylipin production after pathogen infection in mosses have been performed.

*Physcomitrella patens* is infected by several broad host range pathogens, including the phytopathogenic bacteria *Pectobacterium carotovorum (P. carotovorum)* and *Pectobacterium wasabiae*, the fungus *B. cinerea* and the oomycetes *Pythium irregular* and *Pythium debaryanum* (Ponce de León, 2011; Ponce de León and Montesano, 2013). After pathogen assault, *P. patens* activates a defense response similar to flowering plants, including accumulation of reactive oxygen species (ROS), reinforcement of the cell wall, localized cell death known as the hypersensitive response, and activation of defense genes (Ponce de León and Montesano, 2013). *P. patens* respond to biotic stress by increasing the endogenous levels of free unsaturated fatty acid, and inducing the expression of genes encoding different PpLOXs, PpAOS, and an oxophytodienoic acid reductase (Ponce de León et al., 2007, 2012; Oliver et al., 2009). These studies suggest that both the 13-LOX and the 12-LOX pathways are activated after pathogen assault. PpLOX1 and both PpLOX1 and PpLOX6 transcript levels increase after *Pythium* and *B. cinerea* infection, respectively, and OPDA concentrations increase in moss tissues infected with both pathogens (Oliver et al., 2009; Ponce de León et al., 2012). Further studies are needed to evaluate the involvement of the other PpLOXs as well as the oxylipins produced in response to pathogen infection. In flowering plants OPDA is active as a defense signal and regulates defense gene expression (Stintzi et al., 2001; Taki et al., 2005; Browse, 2009). OPDA may play similar roles in mosses since expression levels of the defense gene encoding a phenylalanine ammonia-lyase increase in *P. patens* tissues treated with this oxylipin (Oliver et al., 2009). Moreover, OPDA is a very active oxylipin with antimicrobial activity against several microbial pathogens (Prost et al., 2005), and can therefore contribute to reduce the pathogen population in moss tissues.

Ppα-DOX transcript levels and activity increase in *P. patens* tissues in response to *P. carotovorum* elicitors and *B. cinerea* (Machado et al., 2015). Ppα-DOX-GUS fused proteins accumulate in leaves and protonemal tissues of elicitors-treated and *B. cinerea*-inoculated plants. A protective role against invading pathogens has been proposed for Ppα-DOX since Ppα-DOX-GUS accumulates in *P. patens* cells surrounding *B. cinerea* infected cells (Machado et al., 2015; Figure 3D); this resembles the expression pattern of *Arabidopsis thaliana* α-DOX1-GUS in cells surrounding tissues infected with *Pseudomonas syringae* (Ponce de León et al., 2002). Under normal growth conditions, Ppα-DOX is expressed in protonemal filaments, in rhizoids, and in axillary hairs where it can function as a permanent protection system (Machado et al., 2015). Moreover, functional studies suggest that Ppα-DOX-derived oxylipins protect tissues against cell death caused by elicitors. While Ppα-DOX disrupted mutant have no phenotype and respond similar to wild-type plants, overexpressing Ppα-DOX or treating plants with α-DOX-derived oxylipins, increase protection against cellular damage caused by *P. carotovorum* elicitors (Machado et al., 2015). *P. patens*- and *A. thaliana*-derived oxylipins involved in plant defense are schematized in Figure 4.

## Oxylipins in Other Bryophytes

Compared to mosses, only few studies on oxylipin profiling and enzymes involved in oxylipin formation have been conducted in other bryophytes. In recent years more studies in the liverwort *M. polymorpha* have been performed due to its phylogenetic position as an earliest diverging clade of land plants and the possibility to generate knockout mutants by homologous recombination (Ishizaki et al., 2013). *M. polymorpha* emits C_8_ volatiles and produces OPDA after mechanical wounding (Kihara et al., 2014; Yamamoto et al., 2015). Arachidonic acid (20:4) and eicosapentaenoic acid (20:5) are essential for C_8_ volatiles production in this bryophyte since a fatty acid desaturase knockout mutant with undetectable levels of 20:4 and 20:5 produce only minimal amounts of the C_8_ volatiles (Kihara et al., 2014). Volatiles, including C_5_, C_6_, and C_10_ oxylipins were also identified in the volatile profile of the liverwort *Chiloscyphus pallidus* (Toyota and Akakawa, 1994). The enzymatic activities of three LOXs of *M. polymorpha* have been characterized *in vitro*, and all of them have 15-LOX activity against 20:4 and 20:5 (Kanamoto et al., 2012). Nine additional putative genes encoding LOX are present in the EST database of *M. polymorpha* (Kihara et al., 2014). Besides, an AOC encoding gene has been identified in *M. polymorpha*, which is induced after wounding and OPDA treatment (Yamamoto et al., 2015). This chloroplastic MpAOC is involved in the synthesis of OPDA from the unstable allene oxide 12,13-EOT. The overexpression of MpAOC leads to higher OPDA content and to smaller liverwort plants (Yamamoto et al., 2015). Thus, OPDA acts as a signaling molecule regulating both development and response to wounding in this liverwort.

## Conclusion

In addition to having polyunsaturated C18 fatty acids, algae and mosses also have large amounts of polyunsaturated C20 fatty acids from which they can produce a broad range of oxylipins. In contrast, flowering plants have lost the C20 pathway in the course of evolution. Studies on moss C20-derived oxylipins are therefore of great interest, and will further extend our understanding of the involvement of these metabolites in plant adaptation to biotic and abiotic stress. At the present time, only a few studies in the moss *P. patens* have demonstrated a function for oxylipin-producing enzymes. PpAOC1 and PpAOC2 are needed for spore maturation and dehiscing of the spore capsules, while Ppα-DOX-derived oxylipins participate in development and defense against bacteria. Functional studies in *P. patens* are needed to gain more insights into the diversity of oxylipin metabolic pathways and their involvement in moss development and defense. The evaluation of disease severity and activation of defense mechanisms against pathogens in *P. patens* mutants, including available mutants in PpHPL, PpAOS1, PpAOS2, PpAOC1, and PpAOC2 will certainly improve our understanding of the role played by oxylipins to cope with pathogens. Studies in other bryophytes like the moss *Ceratodon purpureus*, and the liverwort *M. polymorpha* (Brücker et al., 2005; Ishizaki et al., 2013), where homologous recombination based gene disruption is feasible, will also provide valuable information on oxylipin biosynthesis, diversity, and plasticity in early divergent land plants. The lack of JA in *P. patens* is intriguing since this is an important hormone in flowering plants for adaptation to stress. The high content of long and very long chain fatty acids and the production of a wider range of oxylipins including C20-derived oxylipins in moss may be an effective and alternative source of bioactive compounds that contribute to the capacities of these organisms to adapt to diverse environments. The use of other oxylipins than jasmonates for defense responses could be a more general mechanism in bryophytes since *M. polymorpha* does not synthesize JA. Besides, JA may have appeared later in the evolution of plants as a hormone to maximize fitness associated to a higher level of complexity. In conclusion, further studies are needed to obtain a more complete scenario with regard to the evolution of the different oxylipin-producing pathways in bryophytes and the role played by the corresponding enzymes in development and adaptation to stress.

### Conflict of Interest Statement

The authors declare that the research was conducted in the absence of any commercial or financial relationships that could be construed as a potential conflict of interest.
